# Specific Targeting and Labeling of Colonic Polyps in *CPC-APC* Mice with Mucin 5AC Fluorescent Antibodies: A Model for Detection of Early Colon Cancer

**DOI:** 10.3390/cimb45040219

**Published:** 2023-04-11

**Authors:** Michael A. Turner, Kristin E. Cox, Shanglei Liu, Nicholas Neel, Siamak Amirfakhri, Hiroto Nishino, Mojgan Hosseini, Joshua A. Alcantara, Amer Ali Abd El-Hafeez, Thinzar M. Lwin, Kavita Mallya, Joseph R. Pisegna, Satish K. Singh, Pradipta Ghosh, Robert M. Hoffman, Surinder K. Batra, Michael Bouvet

**Affiliations:** 1 Division of Surgical Oncology, Department of Surgery, University of California San Diego, La Jolla, CA 92037, USA; 2 Department of Surgery, VA San Diego Healthcare System, La Jolla, CA 92161, USA; 3 Department of Pathology, University of California San Diego, La Jolla, CA 92037, USA; 4 Department of Cellular and Molecular Medicine, University of California San Diego, La Jolla, CA 92037, USA; 5 Department of Surgical Oncology, City of Hope National Medical Center, Duarte, CA 91010, USA; 6 Department of Biochemistry and Molecular Biology, University of Nebraska Medical Center, Omaha, NE 68198, USA; 7 Department of Gastroenterology, VA Los Angeles Healthcare System, Los Angeles, CA 90073, USA; 8 Medical Service, Section of Gastroenterology, VA Boston Healthcare System, Boston, MA 02130, USA; 9 Department of Medicine, Section of Gastroenterology, Boston University School of Medicine, Boston, MA 02118, USA; 10 Department of Medicine, University of California San Diego, La Jolla, CA 92037, USA; 11 AntiCancer, Inc., San Diego, CA 92111, USA

**Keywords:** colorectal cancer, polyps, fluorescence, fluorescence labeling, mucin, detection, genetically engineered mouse models

## Abstract

Poor visualization of polyps can limit colorectal cancer screening. Fluorescent antibodies to mucin5AC (MUC5AC), a glycoprotein upregulated in adenomas and colorectal cancer, could improve screening colonoscopy polyp detection rate. Adenomatous polyposis coli flox mice with a *Cdx2-Cre* transgene (*CPC-APC*) develop colonic polyps that contain both dysplastic and malignant tissue. Mice received MUC5AC-IR800 or IRdye800 as a control IV and were sacrificed after 48 h for near-infrared imaging of their colons. A polyp-to-background ratio (PBR) was calculated for each polyp by dividing the mean fluorescence intensity of the polyp by the mean fluorescence intensity of the background tissue. The mean 25 μg PBR was 1.70 (±0.56); the mean 50 μg PBR was 2.64 (±0.97); the mean 100 μg PBR was 3.32 (±1.33); and the mean 150 μg PBR was 3.38 (±0.87). The mean PBR of the dye-only control was 2.22 (±1.02), significantly less than the 150 μg arm (*p*-value 0.008). The present study demonstrates the ability of fluorescent anti-MUC5AC antibodies to specifically target and label colonic polyps containing high-grade dysplasia and intramucosal adenocarcinoma in *CPC-APC* mice. This technology can potentially improve the detection rate and decrease the miss rate of advanced colonic neoplasia and early cancer at colonoscopy.

## 1. Introduction

Colorectal cancer (CRC) is the second leading cause of cancer-related deaths in the United States [1]. Fortunately, the mortality of colon cancer is preventable if it is detected early, especially if the colon cancer is found as a premalignant or malignant polyp without any lymph node or metastatic spread [2]. For example, early colorectal cancer (T1) is associated with a much higher 5-year survival rate of 91% compared to 14% if there is metastatic spread. Yet despite knowing this, the overall survival of patients with colon cancer at 5 years is still only 65% due to its typically advanced stage at diagnosis [3].

Colonic mucosal cells can undergo a series of malignant transformations via the tubular adenoma (TA) or the sessile serrated adenoma (SSA) pathways to form precancerous polyps, which progress to colorectal cancer [4,5,6,7,8]. TAs exhibit cytological dysplasia and are premalignant lesions accounting for 65–70% of CRCs, while the SSA is known to have architectural dysplasia and accounts for 15–30% of CRCs [9].

Current clinical guidelines use colonoscopy as the gold standard for colon cancer screening for all patients ages 45 or older, with screening beginning earlier if there is a positive family history [10]. Even if an alternative modality of screening is used (i.e., fecal testing of tumor DNA, fecal occult blood testing, CT colonography, etc.), a positive result still leads to evaluation via colonoscopy. Accurate identification of premalignant polyps during colonoscopy allows for endoscopic excision, which is both diagnostic and therapeutic in preventing further malignant transformation. However, despite improvements in endoscopic technology, the miss rate for precancerous colon adenomas can still be as high as 9% for TA and 27% for SSA, probably due to its flat mucosal appearance hindering detection [11,12,13,14]. This issue is further complicated by the fact that many sessile lesions, such as hyperplastic polyps (HP), are benign and nearly indistinguishable from the more dangerous SSA, which accounts for only 20–30% of sessile polyps [7,15].

Hence, the problem can be summarized by two important features: (1) the fear of missing dangerous colonic polyps, including TAs and SSAs, and (2) the high false positive rate during colonoscopy. These conspire to waste significant medical resources on biopsies of benign polyps without providing a meaningful improvement in colon cancer detection rates. For these reasons, newer detection methodologies are urgently needed for colorectal cancer screening.

The technology surrounding colonoscopy has advanced in recent decades to address this issue. One such tool that has emerged is narrow-band imaging (NBI), which allows the microvascular architecture to be visualized by applying specialized filters [16,17,18,19,20]. Unfortunately, multiple studies have concluded that narrow-band imaging does not improve polyp detection rates [21,22]. Sabbagh et al. did, however, show that detection of benign HPs was reduced using narrow band imaging with 30.1% of polyps in the NBI group versus 41.6% of polyps in the conventional group being HPs with a *p*-value of 0.009. This is important as reduced detection and removal of benign HPs can help to reduce wasted medical resources. However, for villous adenomas, tubulovillous adenomas, or even adenocarcinoma, no improved detection was seen with NBI [21].

Another tool for improved detection is chromoendoscopy (CE). This technology utilizes contrast or absorptive stains that are applied directly to the mucosa during conventional endoscopy [23,24]. The most used stains are indigo carmine (contrast) and methylene blue (absorptive). In a review, van den Broek et al. evaluated three randomized control trials comparing chromoendoscopy to conventional colonoscopy [25]. The first, Brooker et al., found improved detection of < 5 mm polyps with CE (*p*-value 0.026) though not for overall detection rates of adenomas (*p*-value 0.06). CE was also associated with longer withdrawal times, however (9:05 vs. 4:52 min) [26]. Hurlstone et al. found increased detection rates with CE (112 vs. 57) though only highly experienced chromoendoscopists participated in the study, which brings to question the value of widespread implementation of this technology to more standard-trained endoscopists [27]. In the final study, Lapalus et al. also only found a significant increase in detection for <5 mm polyps [28].

Despite these advances in technology for screening colonoscopies, improved detection of premalignant and malignant lesions has yet to be consistently shown. The present study details a novel method of enhancing polyp detection during endoluminal view using targeted fluorescence. The basis of this uses a modified model of the *APC* mouse, termed *CPC-APC*, developed by Hinoi et al., in which mice develop spontaneous colonic polyps that contain both dysplastic and malignant tissue [29]. This model produces polyps that are analogous to the tumorigenesis seen in colorectal cancer with the deletion of APC gene by a Cre recombinase under the control of a colon-specific (CDX2) promotor.

Previously, our laboratory has demonstrated the ability to label orthotopic xenograft mouse models of colon cancer with a mucin 4 antibody conjugated to a near-infrared (NIR) fluorophore [30]. The mucin family of glycoproteins contains twenty-four mucin proteins (MUC1–MUC24) which are involved in cell signaling and barrier protection [31]. Mucins have been shown to have diverse expression profiles among numerous organ systems and between normal vs. premalignant vs. malignant tissues within the colon [32]. MUC4 is known to be expressed within the normal colon [33,34], while MUC5AC is absent [35,36,37], making it a superior target for the detection of premalignant and malignant colonic tissues. MUC5AC has been shown to be expressed in ~50% of colorectal cancers (CRCs), while MUC4 overexpression is only seen in ~25% of CRCs [38,39]. MUC5AC is also more commonly expressed in poorly differentiated CRCs compared to well-to-moderately differentiated CRCs [40].

MUC5AC is a promising marker for the identification of polyps undergoing malignant transformation, as it has been shown to be a distinguishing feature between SSA and HP polyps. MUC5AC has been reported to be expressed in 11.1–43.4% of benign hyperplastic polyps, while two independent groups have both reported its expression in 61% of sessile serrated adenomas [41,42]. The absence of MUC5AC expression within the normal colon, its overexpression in 50% of CRCs, and its ability to aid in distinguishing HPs from SSAs, make MUC5AC a potentially superior target to MUC4 for labeling premalignant and malignant polyps. We have previously demonstrated the ability of MUC5AC-IR800 to label pancreatic cancers and in the present study explore its application within colorectal cancer [43]. The present work demonstrates the use of MUC5AC conjugated with a NIR fluorescent dye for specific targeting of mixed dysplastic-malignant polyps in the CPC-APC mouse model.

## 2. Materials and Methods

### 2.1. Antibody Conjugation

Monoclonal mucin 5AC antibody (45M1, Novus Biologicals, Littleton CO, USA) was conjugated to the near-infrared (NIR) dye IRDye800CW NHS ester (LI-COR Biosciences, Lincoln, NE, USA), establishing MUC5AC-IR800. The dye was conjugated to the antibody per the manufacturer’s protocol and incubated at room temperature for 2 h on a shaker plate. After incubation, the antibody-dye conjugate was added to gel-desalting columns (Thermo Fisher Scientific, Waltham, MA, USA) to remove the excess unbound dye. The final product was stored at 4 °C.

### 2.2. Mouse Models

The CPC-APC mouse model [29] is a conditional knockout of the adenomatous polyposis coli (APC) gene, which spontaneously develops distal colorectal polyps at approximately 10 weeks of age. The experimental genotype CDX2-Cre+; APC^flox/+^ is generated by crossing C57BL/6_CDX2-Cre+ with C57BL/6_APC^flox/+^ or C57BL/6_APC^flox/flox^. Ear tissue samples were collected on day 28 for genotyping. Mice were closely monitored for complications associated with the development of polyps: anal bleeding, anal prolapse, weight loss, and lethargy. Moribund mice and mice that lost >15% of body weight were euthanized. All animal breeding and experiments were approved and conducted under protocol S18086 in accordance with Institutional Animal Care and Use Committee (IACUC) guidelines at the University of California, San Diego. Mice were caged in groups of 1–5 in a HEPA-filtered facility and fed a standard autoclaved diet.

### 2.3. Antibody-Conjugate Administration and Imaging

Mice were randomized into 4 different dose arms of MUC5AC-IR800: 25 μg (*n* = 3), 50 μg (*n* = 3), 100 μg (*n* = 3), and 150 μg (*n* = 3). The control (*n* = 2) mice received dye-only injections. To determine the amount of dye for the control arm, the moles of dye in the 150 μg arm were calculated, and the control arm received an equivalent dose of IRDye800. The mice were anesthetized with an intraperitoneal injection of a solution of xylazine, ketamine, and phosphate-buffered saline (PBS). They were placed on a heating pad at 35 °C for approximately 5 min to encourage vasodilation of their tail veins. The antibody-dye conjugate, or the control, was then administered via tail vein injection.

After 48 h, the mice were euthanized by CO_2_ inhalation, which was then confirmed with cervical dislocation. The colon and rectum were removed, opened, placed on a petri dish, and imaged with the Pearl Trilogy Small Animal Imaging System (LI-COR, Lincoln, NE, USA) using a 767 nm excitation and generating a 786 nm emission (more information at Licor.com/bio/reagents product number 929-72020). For each mouse, every polyp was designated as a region of interest (ROI), and the Pearl Trilogy Small Animal Imaging System was used to calculate the mean fluorescence intensity (mFI) for each ROI. To calculate a background tissue mFI for each mouse, five ROIs over normal tissue were selected, and the average was used as the background mFI. Each polyp had a polyp-to-background ratio (PBR) calculated by dividing the mFI of the polyp by the average background tissue mFI for that colon. The mean PBR and standard deviation for each MUC5AC-IR800 dose arm were calculated from each polyp in that arm.

### 2.4. Histology

Polyps were placed in formalin solution for 72 h before being placed in paraffin for sectioning. The H&E stained slides were read by a senior pathologist (MH) and reviewed by the first author (MAT).

### 2.5. Statistical Analysis

Statistical analysis was performed with R software (Free Software Foundation, Boston, MA, USA). Analysis of Variance (ANOVA) statistical analysis with the Tukey method was used to assess the statistical difference between the 4 doses. The Student’s *t*-test was used to evaluate the statistical difference between the 150 μg and control arms.

## 3. Results

### 3.1. Specific Labeling of Colonic Polyps with MUC5AC-IR800

**Figure 1:**
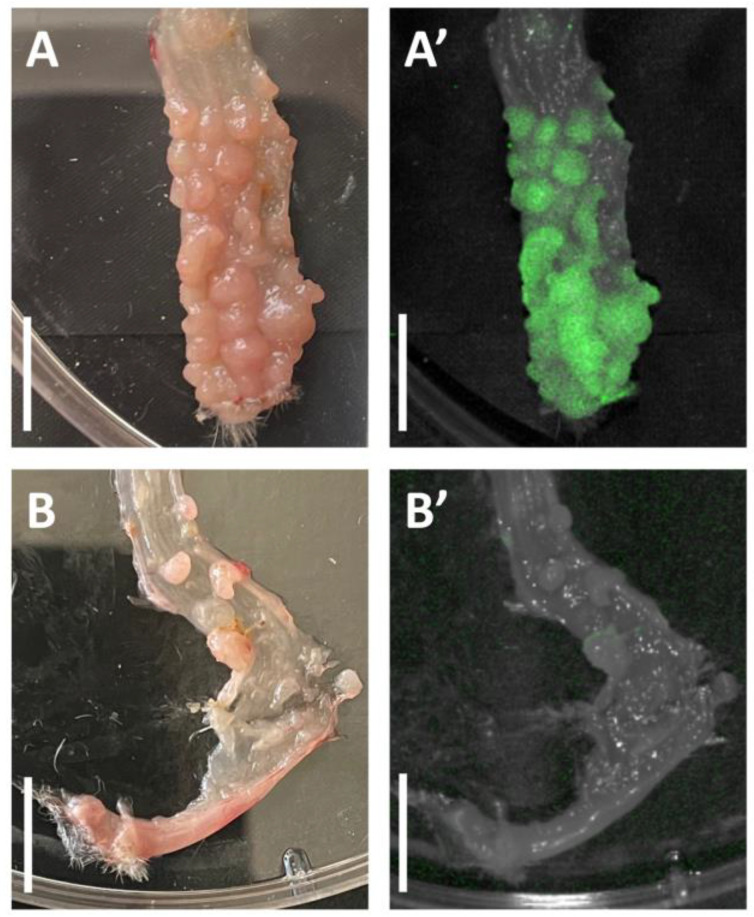
*CPC-APC* mouse colon (rectum at the bottom of image) opened longitudinally and imaged 48 h after administration of 150 µg MUC5AC-IR800 (panel (**A**) = bright light, panel (**A’**) = NIR imaging) with specific labeling of polyps in NIR image. Compared to CPC-APC mouse colon opened longitudinally and imaged 48 h after administration of comparable moles of NIR dye as a control (panel (**B**) = bright light, panel (**B’**) = NIR imaging). Scale bar: 1 cm.

*CPC-APC* mice received an intravenous injection of 150 µg MUC5AC-IR800 or equivalent amounts of IRDye800 as a control 48 h prior to imaging. Following euthanasia, the colons were removed and opened to allow intra-luminal imaging. The mice that received 150 µg MUC5AC-IR800 had specific labeling of the polyps, while the control had low levels of non-specific fluorescence (Figure 1).

### 3.2. Increased Polyp to Background Ratios with Higher Dosage of MUC5AC-IR800

**Figure 2:**
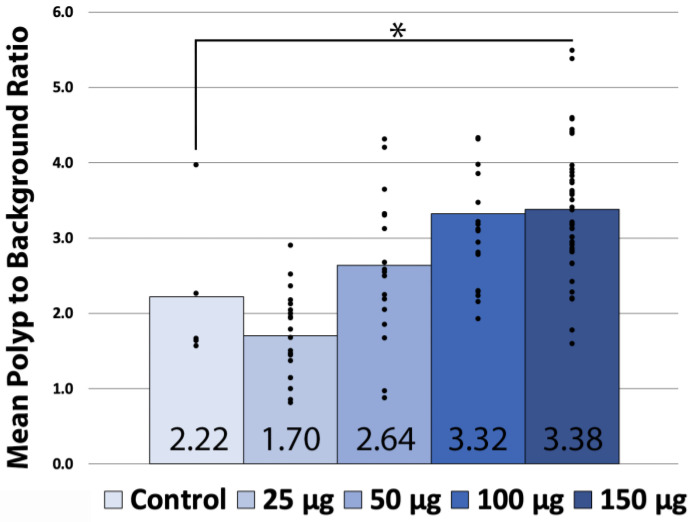
Mean polyp to background ratios in *CPC-APC* colonic polyps 48 h after administration of various dosages (25 µg, 50 µg, 100 µg, and 150 µg) of MUC5AC-IR800 or IRDye800 as a control. Dots represent individual PBRs within the treatment group. * = *p*-value 0.008.

The mean polyp background ratio (PBR) for the mice treated with 25 μg of MUC5AC-IR800 was 1.70 (±0.56). The mice treated with 50 μg of MUC5AC-IR800 had a mean PBR of 2.64 (±0.97). The mice treated with 100 μg of MUC5AC-IR800 had a mean PBR of 3.32 (±1.33). Mice treated with 150 μg of MUC5AC-IR800 had a mean PBR of 3.38 (±0.87). Mice in the control group had a mean PBR of 2.22 (±1.02) (Figure 2).

### 3.3. Fluorescence Intensity

**Figure 3:**
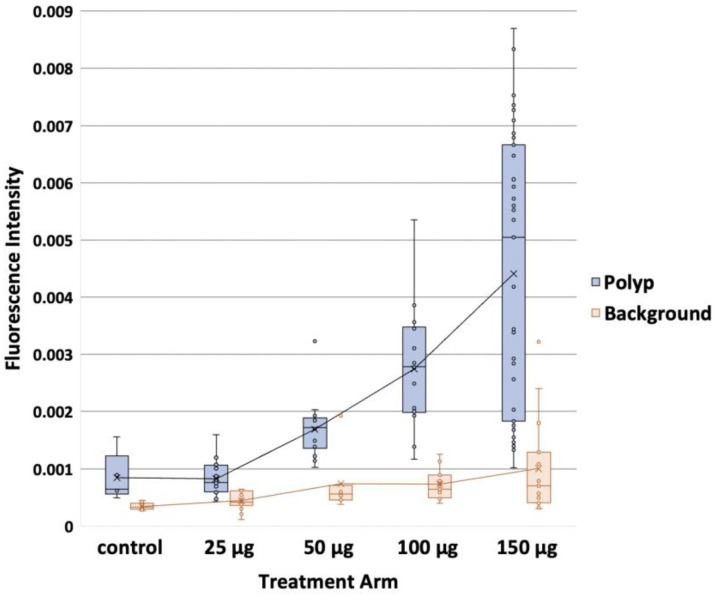
Box plots of polyp and background tissue fluorescence intensity for *CPC-APC* mice 48 h after administration of various doses (25 µg, 50 µg, 100 µg, and 150 µg) of MUC5AC-IR800 or IRDye800 as control. Mean values are denoted by x within box plots with the mean line is shown.

Despite the increasing dosage of MUC5AC-IR800, the background fluorescence intensities remained relatively constant among the treatment groups (Figure 3). Polyp fluorescence intensity, however, increased with increasing dose of MUC5AC-IR800. Polyps in the 150 µg MUC5AC-IR800 group had a mean fluorescence intensity 2.6 times greater than the mean fluorescence intensity of polyps in the 50 µg MUC5AC-IR800 group. For the control, the polyp fluorescence intensity was three to five times lower than the 100 µg or 150 µg MUC5AC-IR800 groups.

### 3.4. Statistical Analysis

An analysis of variance (ANOVA) was performed for the 25 μg, 50 μg, 100 μg, and 150 μg cohorts. An F-value of 13.26 (*p*-value < 0.001) was calculated, indicating a significant difference in the mean PBR of the arms. Tukey honestly significant difference (HSD) showed a significant difference between all doses except 50 μg: 100 μg and 100 μg: 150 μg (Table 1). A Student’s *t*-test was used to compare the mice treated with 150 μg of MUC5AC-IR800 and the control arm, which received equivalent moles of dye. The student’s *t*-test showed a significant difference between the two groups (*p*-value 0.008) (Figure 2).

### 3.5. Polyps Contain Dysplasia and Adenocarcinoma

**Figure 4:**
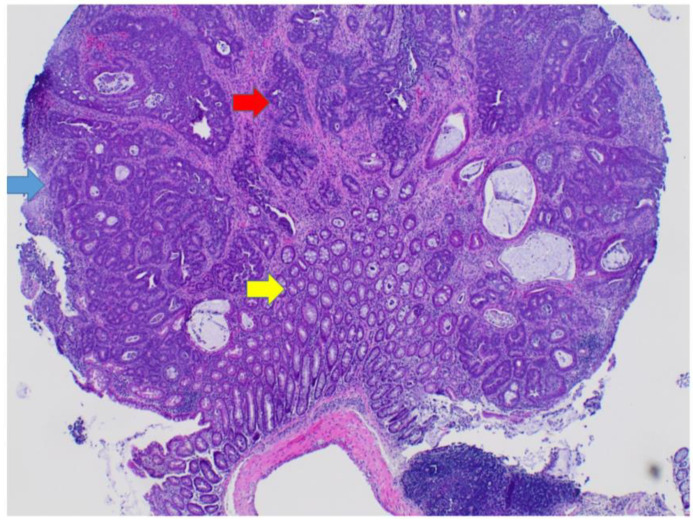
H&E staining demonstrating high-grade dysplasia (blue arrow) and intramucosal adenocarcinoma (red arrow) within the murine polyp without invasion of the basement membrane. Yellow arrow indicates normal murine colonic tissue.

Hematoxylin and eosin (H&E) staining of polyps demonstrated areas of high-grade dysplasia as well as intramucosal adenocarcinoma (Figure 4), demonstrating that the *CPC-APC* model produces analogous tumorigenesis of colorectal cancer in the observed polyps.

## 4. Discussion

The present proof-of-concept study demonstrates the ability of MUC5AC-IR800 to selectively label colorectal polyps in *CPC-APC* mice compared to NIR dye alone. This is an important preclinical step in integrating this technology to increase polyp identification during intraluminal evaluation (i.e., screening colonoscopies). Screening colonoscopies and polypectomies are associated with decreased rates of colorectal cancer (CRC) and deaths from CRC [44,45]. However, this technique is plagued with a high miss rate for adenomas (10–22% [11,46]) and CRC (1.8–5.9% [47,48]). In a population-based case-controlled review of interval cancers after a negative colonoscopy, interval cancer cases were more likely to be in the proximal colon, more likely to have had an incomplete colonoscopy (failure to reach the cecum), and more likely to have had a colonoscopy performed for a positive fecal occult blood test rather than primary screening when compared to control cases [49]. Given these findings and the rate of interval cancers that occurred < 3 years after a negative colonoscopy, the authors conclude these interval cancers most likely represented missed neoplasms. A similar study looking only at new cancer diagnoses within 3 years of a negative colonoscopy found an interval cancer rate of 3.44% [47]. The current approach to screening colonoscopies, relying heavily on physician attentiveness and visual cues for the successful identification of suspicious lesions, needs to be augmented [46].

Conversely, if diminutive (<5 mm) polyps are identified during colonoscopy, many of them have benign pathology and pose little to no malignant potential. Because of this, many clinicians have proposed a pluck-and-discard strategy to avoid wasting medical resources for pathologic analysis [50,51]. However, this method has not yet become common practice due to the fear of missing small malignant polyps. Fluorescence identification of polyps provides another source of malignant polyp discrimination based on differences in protein expression as a polyp undergoes malignant transformation.

MUC5AC was chosen as the molecular target in this study, given its upregulation in CRC and malignant polyps [36]. MUC5AC is normally absent in colorectal mucosa but has de novo expression in CRC progression [52,53]. Increased MUC5AC expression has also been associated with microsatellite instability (MSI) and proximal colon polyp location, both characteristics associated with missed CRCs on colonoscopy [54,55,56]. Furthermore, one of the key features of MUC5AC that distinguishes it from other CRC markers is its expression in SSA, which has a much higher malignant potential than their nearly visually identical HP [57]. The successful differentiation of SSA from HP during a visual inspection at the time of polypectomy is key to developing an efficient clinical protocol for polyp management, as the majority of polyps removed are benign and do not require full pathological analysis. The antibody to human MUC5AC used in the present study has cross-reactivity with murine MUC5AC [58]. This is important as we could expect translatability of these results clinically.

While there is no accepted PBR value that is considered “positive” in the literature, the PBRs in the present study ranged from 1.70 to 3.38, which provides enough contrast between polyps and surrounding tissue to aid in detection. Recent clinical trials with fluorescence guided surgery (FGS) showed that an intraoperative tumor-to-background ratio of 1.6 [59], 1.83 [60], and 1.9 [61] was sufficient to detect the tumor of interest. Keller et al. reported a clinical trial of 27 patients where an anti-CEA fluorescent antibody was applied topically to large colonic polypoid lesions enabled improved distinction of polypoid tissue from benign mucosa [62]. One obvious benefit to the present approach is that MUC5AC-IR800 is injected systemically, obviating the need for initial white light detection and then local application, as suggested by Keller et al. [62]. This is an important distinction that allows probes such as MUC5AC-IR800 to become an aid for screening as it does not rely on endoscopists’ ability to identify suspicious areas that would prompt its application.

One limitation of the proposed approach is the possible decreased signal penetration in the presence of bleeding, mucosal capping, or other visually obstructive particles during evaluation. Further research is needed to evaluate the effect of bleeding, ulceration, or general inflammation (e.g., colitis) on MUC5AC-IR800′s ability to differentiate polyps from abnormal, yet benign colonic tissue. An additional limitation of the present study includes the measurement of fluorescence signal occurred ex vivo. Although this is an active area of development, a commercially available flexible endoscope that allows for NIR in the 800 nm wavelength range is not yet available, though prototypic systems have been developed for NIR colonoscopy [63]. Burggraff et al. performed a pilot study in humans with an anti-c-MET antibody conjugated to Cy5 (emissions within the near-infrared spectra). With the use of fluorescence, they were able to identify an additional 9 polyps that were not detected with white light alone. However, their probe detected benign hyperplastic polyps in addition to adenomas.

The present study demonstrates the ability of MUC5AC-IR800 to specifically target and label colonic polyps containing high-grade dysplasia and intramucosal adenocarcinoma in *CPC-APC* mice. Future studies are needed to determine the polyp-to-background ratio for benign polyps in these mice. We plan to perform similar experiments at earlier time points prior to the development of carcinoma. Once MUC5AC-IR800 can be confirmed to have high specificity and sensitivity for premalignant and malignant polyps (or possibly MUC5AC-IR800 in combination with another antibody targeting a specific subset of premalignant or malignant tissues), the present technique can be used for improved detection of polyps during colonoscopy. No toxicity was observed in the mice following MUC5AC-IR800 administration at any dose. While further studies are needed to identify the optimal timing, dosing, and tolerability of the antibody in humans, this proof-of-concept study is an important preclinical step. Additional molecular targets could also be identified and evaluated using the same principles of the present study. To have maximal clinical utility, the targets would need to be specific to the malignant transformation of polyps to increase the specificity of distinguishing cancerous and precancerous polyps from their benign counterparts. Improving polyp detection and removal rate in screening colonoscopies may decrease the miss rate of malignant polyps and lead to lower incidence and, thus, death from CRC.

## Figures and Tables

**Table 1:** ANOVA with Tukey HSD (honestly significant difference) comparing the difference of mean PBR among the four treatment doses.

Dose Comparison	Difference in Mean PBR	*p*-Value
25 µg–50 µg	0.934 (0.096–1.772)	0.021 *
25 µg–100 µg	1.622 (0.784–2.460)	<0.001 **
25 µg–150 µg	1.682 (0.988–2.377)	<0.001 **
50 µg–100 µg	0.688 (−0.182–1.558)	0.189
50 µg–150 µg	0.748 (0.016–1.481)	0.043 *
100 µg–150 µg	0.060 (−0.672–0.793)	0.999

* = *p*-value < 0.05. ** = *p*-value < 0.01

## Data Availability

Not applicable.
